# Impact of Amino Acid Substitutions in B Subunit of DNA Gyrase in *Mycobacterium leprae* on Fluoroquinolone Resistance

**DOI:** 10.1371/journal.pntd.0001838

**Published:** 2012-10-11

**Authors:** Kazumasa Yokoyama, Hyun Kim, Tetsu Mukai, Masanori Matsuoka, Chie Nakajima, Yasuhiko Suzuki

**Affiliations:** 1 Division of Global Epidemiology, Hokkaido University Research Center for Zoonosis Control, Sapporo, Hokkaido, Japan; 2 Leprosy Research Center, National Institute of Infectious Diseases, Higashimurayama, Tokyo, Japan; 3 JST/JICA-SATREPS, Tokyo, Japan; Fondation Raoul Follereau, France

## Abstract

**Background:**

Ofloxacin is a fluoroquinolone (FQ) used for the treatment of leprosy. FQs are known to interact with both A and B subunits of DNA gyrase and inhibit supercoiling activity of this enzyme. Mutations conferring FQ resistance have been reported to be found only in the gene encoding A subunit of this enzyme (*gyrA*) of *M. leprae*, although there are many reports on the FQ resistance-associated mutation in *gyrB* in other bacteria, including *M. tuberculosis*, a bacterial species in the same genus as *M. leprae*.

**Methodology/Principal Findings:**

To reveal the possible contribution of mutations in *gyrB* to FQ resistance in *M. leprae*, we examined the inhibitory activity of FQs against recombinant DNA gyrases with amino acid substitutions at position 464, 502 and 504, equivalent to position 461, 499 and 501 in *M. tuberculosis*, which are reported to contribute to reduced sensitivity to FQ. The FQ-inhibited supercoiling assay and FQ-induced cleavage assay demonstrated the important roles of these amino acid substitutions in reduced sensitivity to FQ with marked influence by amino acid substitution, especially at position 502. Additionally, effectiveness of sitafloxacin, a FQ, to mutant DNA gyrases was revealed by low inhibitory concentration of this FQ.

**Significance:**

Data obtained in this study suggested the possible emergence of FQ-resistant *M. leprae* with mutations in *gyrB* and the necessity of analyzing both *gyrA* and *gyrB* for an FQ susceptibility test. In addition, potential use of sitafloxacin for the treatment of problematic cases of leprosy by FQ resistant *M. leprae* was suggested.

## Author Summary

Leprosy is one of the oldest human infectious diseases, which remains a public health problem with more than 200,000 new cases every year worldwide. Since the late 1990s, multi-drug resistant leprosy, resistant to rifampicin and dapsone, has emerged and the importance of ofloxacin has increased. However, their use for leprosy and other infectious diseases has already elicited ofloxacin resistant leprosy cases. Hence, early detection of ofloxacin resistance is essential for proper treatment. This study, by utilizing recombinant technology, predicted the future emergence of ofloxacin resistant *Mycobacterium leprae* with mutations that have not yet been reported. The data are useful for predicting ofloxacin resistance and, hence, able to contribute to the proper treatment of leprosy through suggesting the importance of analyzing gene mutations for FQ susceptibility testing.

## Introduction

Leprosy is one of the oldest human infectious diseases and remains a public health problem. At the beginning of 2011, the number of registered leprosy cases was 192,246, and that of new cases reported during 2010 was 228,474, mainly from Asian, Latin American, and African countries [1]. Multibacillary leprosy is usually treated by administering dapsone (DDS), clofazimine (CLF), and rifampicin (RIF) in combination, where single skin lesion paucibacillary leprosy is recommened to be treated by administering RIF, ofloxacin (OFX), and minocycline (MIN) [2]. Since the late 1990s, multi-drug resistant (MDR) isolates of *M. leprae*, resistant to RIF and DDS, have emerged and the importance of OFX has been a focus for the treatment of MDR-leprosy [3]; however, their use not only for leprosy but also for other infectious diseases including tuberculosis has already led to OFX resistance in *M. leprae* [4]–[8]. Hence, early prediction of FQ resistance seems to be essential for the proper treatment of leprosy.

**Figure 1:**
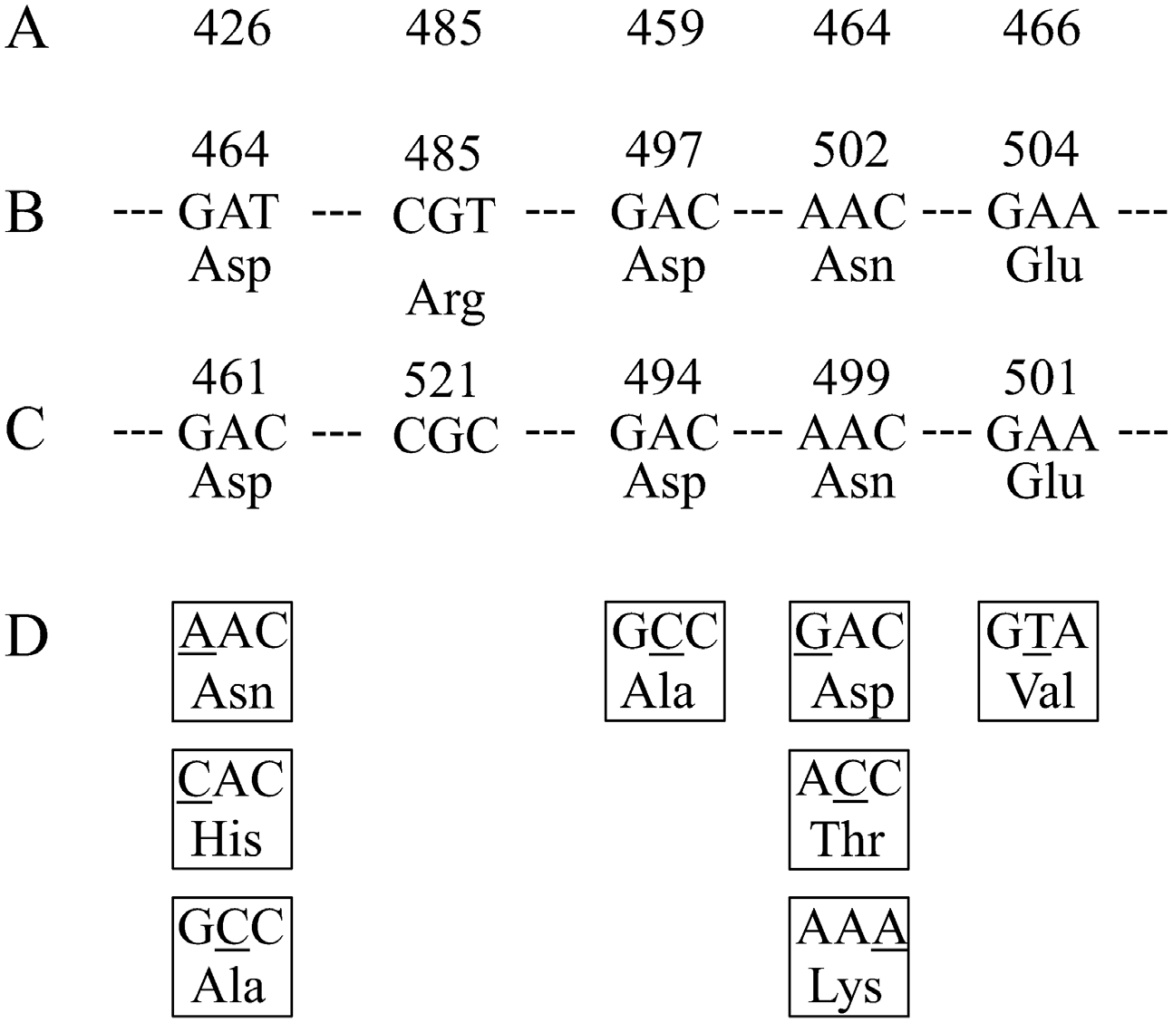
Nucleotide and amino acid sequences of QRDR of *M. leprae* and *M. tuberculosis gyrB* and mutations found in FQ-resistant isolates. (A) amino acid number of GyrB in *E. coli*, (B) Amino acid number, nucleotide sequences and amino acid sequence of WT *M. leprae* GyrB QRDR, (C) Amino acid number, nucleotide sequences and of WT *M. tuberculosis* GyrB QRDR, (D) Altered amino acids and corresponding nucleotide substitutions found in higher rate in FQ-resistant *M. tuberculosis* isolates.

OFX is a fluoroquinole (FQ) and FQs inhibit type II DNA topoisomerases, including DNA gyrase and topoisomerase IV [9]. FQ resistance is given mainly by amino acid substitutions in the quinolone resistance-determining regions (QRDRs) located on the N- and C-terminal domains of A (GyrA) and B (GyrB) subunits of DNA gyrase and, less prominently, amino acid substitution in the QRDR on the N- and C-terminal domains of A (ParC) and B (ParE) subunits of topoisomerase IV has been reported [10]. *M. leprae* has only DNA gyrase [11], which is therefore the sole target of FQs. Genetic analysis of *M. leprae* clinical isolates revealed reduced FQ sensitivity associated with amino acid substitutions only at position 89 or 91 and 205 in GyrA and GyrB, respectively [4]–[8], [12]. In the latter study, the contribution of amino acid substitution in GyrA at position 89 or 91 to reduced FQ sensitivity was confirmed by an *in vitro* analysis [13]. In addition, the effect of amino acid substitution at position 95 in GyrA was predicted [14]. In contrast, amino acid substitution in GyrB at position 205, reported by You et al. [8], was revealed not to affect FQ sensitivity by an *in vitro* study [13]. Reduced FQ sensitivity associated with amino acid substitutions has been frequently reported in GyrA in *M. tuberculosis*; however, those in GyrB have been reported less frequently (Figure 1) [10], [15]. According to the reports, important residues of GyrB in *M. tuberculosis* were thought to be at codon 461, 499 and 501 (with a counting system proposed by Maruri et al. [10]). Notably, amino acid substitutions at position 499 and 501 in *M. tuberculosis* showed a correlation with reduced FQ susceptibility by an *in vitro* assay [15]–[18]. Lack of the detection of FQ-resistant *M. leprae* carrying GyrB amino acid substitutions is due to the low number of FQ resistant cases analyzed. Hence, it is highly important to elucidate the contribution of amino acid substitutions in GyrB to FQ resistance utilizing recombinant technology and *in vitro* assay.

On the basis of reports on *M. tuberculosis*, we selected target amino acid substitutions at position 464, 502 and 504 in *M. leprae* GyrB, equivalent to position 461, 499 and 501 in *M. tuberculosis*, to reveal the significance of these amino acid substitutions for reduced FQ sensitivity, and conducted the FQ-inhibited supercoiling assay and FQ-mediated DNA cleavage assay using recombinant DNA gyrase.

## Methods

### Drugs and kits

Ofloxacin (OFX), ciprofloxacin (CIP) and levofloxacin (LVX) were purchased from LKT Laboratories, Inc. (St. Paul, MN); moxifloxacin (MXF) was from Toronto Research Chemicals Inc. (Toronto, Ontario, Canada); sitafloxacin (SIT) was from Daiichisankyo Pharmaceutical, Co., Ltd. (Tokyo, Japan); ampicillin and kanamycin were purchased from Meiji Seika Pharma Ltd. (Tokyo, Japan). Oligonucleotide primers were synthesized by Life Technologies Corp. (Carlsbad, CA). Restriction enzymes were obtained from New England Biolabs, Inc. (Ipswich, MA). The supercoiling assay kit and supercoiled and relaxed pBR322 DNA were purchased from John Innes Enterprises Ltd. (Norwich, United Kingdom).

### Bacterial strains and plasmid

The Thai-53 strain of *M. leprae* [19], maintained at the Leprosy Research Center, National Institute of Infectious Diseases (Tokyo, Japan), was used to prepare *M. leprae* DNA. *Escherichia coli* strains TOP-10 (Life Technologies Corp.), Rosetta-gami 2, and BL21 (DE3) pLysS (Merck KGaA, Darmstadt, Germany) were used for cloning and protein expression. pET-20b (+) (Merck KGaA) vector was used to construct expression plasmids for *M. leprae* DNA gyrases.

### Construction of expression plasmids

**Figure 2:**
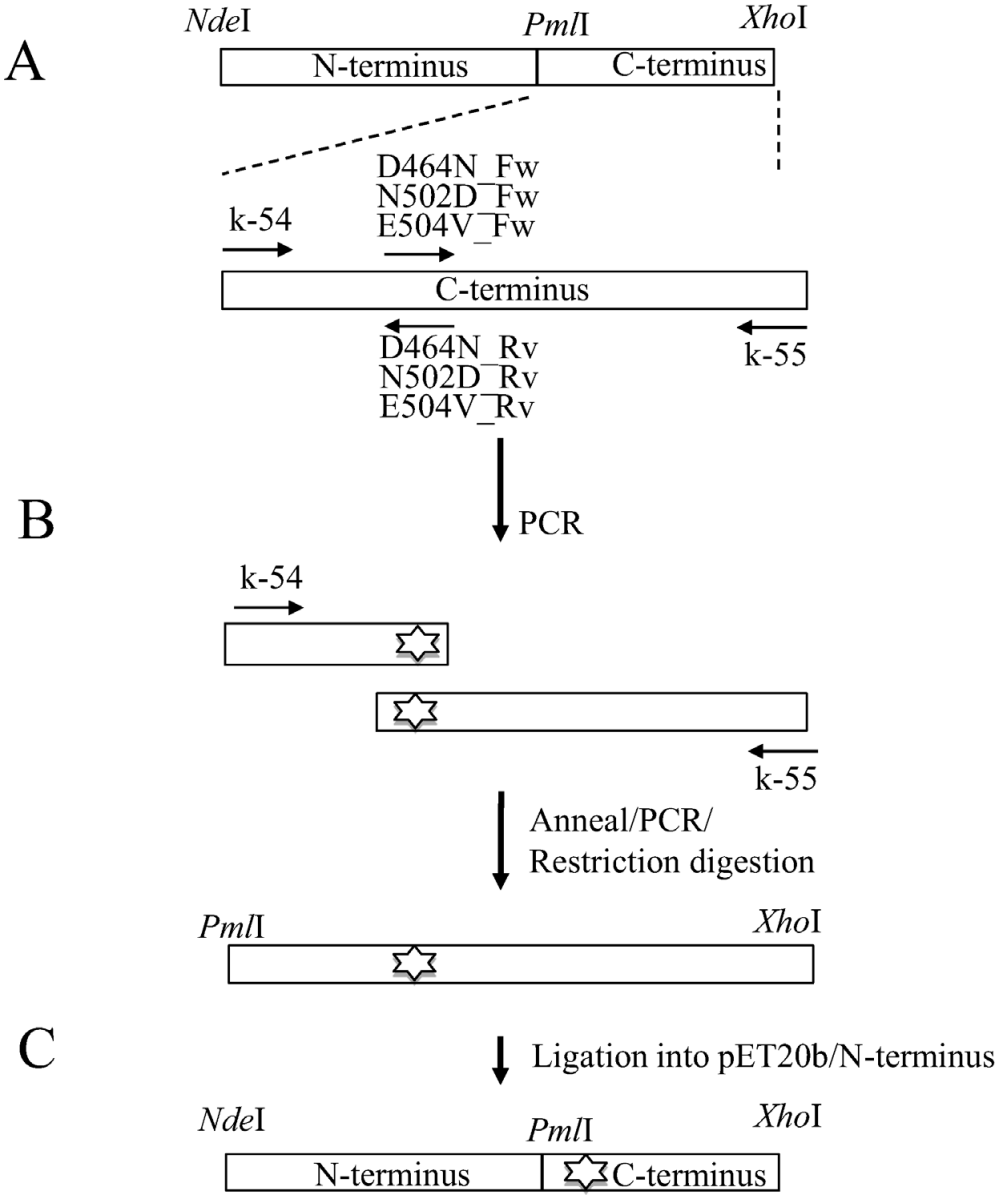
Construction of WT and mutant DNA gyrase expression plasmid. (A) Primer pairs k-54+D464N_Rv, N502D_Rvor E504V_Rv (Table 1) were used for amplifying the DNA fragment encoding N-terminus half (amino acid 424 to 467, 505 or 507, respectively) of C-terminus region of GyrB carrying Asp464Asn, Asn502Asp and Glu504Val, respectively. Primer pairs k-55+D464N_Fw, N502D_Fw or E504V_Fw (Table 1) were used for amplifying the DNA fragment encoding the C-terminus half (amino acid 461, 499 or 501 to 678, respectively) of the C-terminus region of GyrB carrying Asp464Asn, Asn502Asp and Glu504Val, respectively. (B) To complete the C-terminus region encoding cassette, DNA fragments encoding the N-terminus half and C-terminus half of the C-terminus region of GyrB were annealed and reamplified by PCR using the primer pair of k-54 and k-55. (C) The mutated *gyrB*-C cassettes were digested with *Pml*I and *Xho*I restriction endonucleases and ligated into the expression plasmid containing the WT*gyrB*N-terminus region DNA fragment digested by the same enzymes.

Wild-type (WT) recombinant GyrA and GyrB expression plasmids were constructed as described previously [14], [16]. Mutations were introduced into the WT *gyrB* gene by PCR using pairs of complementary primers containing the mutations of interest (Table 1). All PCR reactions were carried out in a thermal cycler (Life Technologies Corp.) under the following conditions: pre-denaturation at 95°C for 2 min; 35 cycles of denaturation at 95°C for 10 s, annealing at 50–60°C for 15 s, and extension at 68°C for 1 to 3 min, and then a final extension at 68°C for 5 min. The *gyrB* C-terminal cassettes with base substitutions were digested with *Pml* I and *Xho* I, ligated into WT *gyrB* expression plasmid, and digested with the same restriction endonucleases to obtain mutant *gyrB* expression plasmid (Figure 2). The nucleotide sequences of the DNA gyrase genes in the plasmids were confirmed using a BigDye Terminator (version 3.1) cycle sequencing kit and an ABI Prism 3130xI genetic analyzer (Life Technologies Corp.) according to the manufacturer's protocol.

**Table 1:** Nucleotide sequences of primers used in this study.

Primer name	Primer sequence (Nucleotide Position)
k-54	5′-CGTAAAGCACGTGAGTTAGTGCGTCGAAAAAGTGCC-3′ (1270–1305)
k-55	5′-GGCTCGAGCTAATGATGATGATGATGATGGACATCCAGGAAACGAACATCC-3′ (2013–2037)
D464N_Fw	5′-A GTG GAA GGT **AAT** TCG GCT GGT G
D464N_Rv	5′-C ACC AGC CGA **ATT** ACC TTC CAC T
N502D_Fw	5′-A GTG CTA AAG **GAC** ACC GAA GTT C
N502D_Rv	5′-G AAC TTC GGT **GTC** CTT TAG CAC T
E504V_Fw	5′-A AAG AAC ACC **GTA** GTT CAA GCA A
E504V_Rv	5′-T TGC TTG AAC **TAC** GGT GTT CTT T

Mutated codons are indicated in bold face.

### Expression and purification of recombinant DNA gyrase subunits

Recombinant DNA gyrase subunits were expressed and purified as previously described [13], [14], [16], [20]. Briefly, expression plasmids carrying the *gyrA* and *gyrB* of *M. leprae* were transformed into *E. coli* Rosetta-gami 2 and BL21 (DE3) pLysS, respectively. The transformants were grown in Luria-Bertani (LB) medium in the presence of 100 µg/mL Ampicillin to the log phase and the expression of DNA gyrase was induced with the addition of 1 mM isopropyl-beta-D-thiogalactopyranoside (Wako Pure Chemical Industries Ltd., Osaka, Japan), followed by further incubation at 14°C for 16 h. The harvested *E. coli* were lysed by sonication (Sonifier 250; Branson, Danbury, CT) and the recombinant DNA gyrase subunits in supernatants after centrifugation (10,000× g for 30 min) were purified by Ni-NTA Agarose resin (Life Technologies Corp.) column chromatography and dialyzed against DNA gyrase dilution buffer (50 mM Tris-HCl pH 7.5, 100 mM KCl, 2 mM DTT, 1 mM EDTA). The purified protein fractions were examined by sodium dodecyl sulfate-polyacrylamide gel electrophoresis (SDS-PAGE).

### DNA supercoiling assay and inhibition by FQs

ATP-dependent and FQ-inhibited DNA supercoiling assays were performed according to previous reports [13], [14], [16], [20]. DNA supercoiling activity was examined with reaction mixture consisting of DNA gyrase reaction buffer, relaxed pBR322 DNA (0.3 µg), and GyrA and GyrB subunits (50 ng each) in a total volume of 30 µl. Reactions were run at 30°C for 1.5 h followed by stopping with the addition of 30 µl chloroform/iso-amyl alcohol (24∶1 mixture) and 3 µl of 10× DNA loading solution. The total reaction mixtures were subjected to electrophoresis on 1% agarose gels in 1× Tris-borate-EDTA (TBE) buffer and stained by ethidium bromide (0.7 µg/ml). The extent of supercoiled DNA was quantified with ImageJ (http://rsbweb.nih.gov/ij) and the inhibitory effects of FQs on DNA gyrase were assessed by determining the drug concentration required to inhibit the supercoiling activity of the DNA gyrase by 50% (IC_50_s) in the presence or absence of serial two-fold increases in the concentrations of OFX, MXF, SIT, CIP and LVX. Enzymatic assays were performed at least three times to confirm the reproducibility.

### FQ-mediated DNA cleavage assay

DNA cleavage assays were also carried out as described in previous reports [13], [14], [16], [20], [21]. Briefly, the reaction mixture (total volume 30 µl) contained DNA gyrase assay buffer, purified DNA gyrase subunits, supercoiled pBR322 DNA (0.3 µg) and increasing concentrations of OFX, MXF, SIT, CIP and LVX. After incubation for 2 h at 30°C, cleavage reactions were stopped by adding 3 µl of 2% SDS and 3 µl proteinase K (1 mg/ml). After subsequent incubation for 30 min at 30°C, proteinase K reactions were stopped by the addition of 3 µl of 0.5 mM EDTA, 30 µl chloroform/iso-amyl alcohol (24∶1 mixture) and 3 µl of 10× DNA loading dye. The total reaction mixtures were subjected to electrophoresis in 0.8% agarose gels in 1× TBE buffer, followed by ethidium bromide staining. The extent of DNA cleavage was quantified with ImageJ (http://rsbweb.nih.gov/ij) and the FQ concentrations required to induce 25% of the maximum DNA cleavage (CC_25_s) were determined.

## Results

### Construction and purification of recombinant WT and mutant DNA gyrase subunits

The WT GyrA and GyrB expression plasmids constructed in our previous work [14] were used. DNA fragments with mutations causing amino acid substitutions at position 464, 502 and 504 in GyrB were amplified from WT GyrB expression plasmid [14] and introduced into expression vector pET-20b (+). Recombinant GyrA and GyrB were expressed as C-terminus hexa-histidine tagged protein for ease of purification, as the His-tag has been shown not to interfere with the catalytic functions of GyrA and GyrB [13]–[16], [20], [22]. Expressed recombinant WT and mutant DNA gyrase subunits were purified as 0.4 to 1.7 mg soluble His-tagged protein with molecular weights of 80 kDa and 75 kDa for GyrA and GyrB, respectively, from 500 ml cultures. The purity of recombinant proteins was confirmed by SDS-PAGE (Figure S1). All of the recombinant proteins were obtained with high purity (>90–95%).

### ATP-dependent DNA supercoiling activities of WT and mutant DNA gyrases

Combinations of WT GyrA and WT or mutant GyrBs (GyrB-Asp464Asn, GyrB-Asn502Asp or GyrB-Glu504Val) were examined for DNA supercoiling activities using relaxed pBR322 DNA as a substrate in the presence or absence of ATP (Figure S2). DNA supercoiling activities were observed in the presence of ATP and recombinant DNA gyrase subunits (Figure S2 A–D, lane 3), while neither subunit alone exhibited DNA supercoiling activity (Figure S2 A–D, lane 4, 5). In addition, no supercoiling activity was observed when ATP was omitted from the reaction condition (Figure S2 A–D, lane 6). Consequently, ATP-dependent DNA supercoiling activities were confirmed with WT and three mutant DNA gyrases.

### IC_50_s of five FQs for WT and mutant DNA gyrases

**Figure 3:**
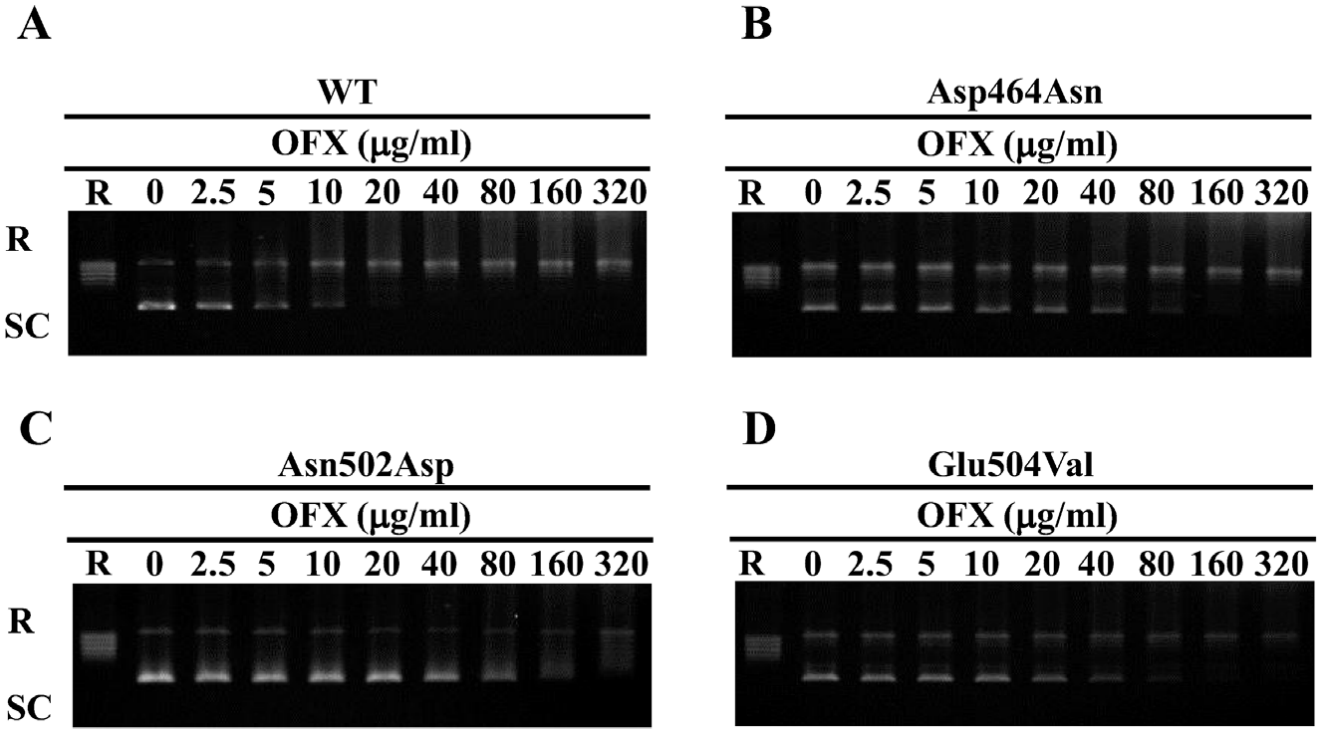
OFX-inhibited DNA supercoiling assay. Relaxed pBR322 (0.3 mg) was incubated with GyrA (50 ng) and GyrB (50 ng) in the presence of the indicated concentration of OFX. FQ-inhibited supercoiling activity assay was performed in combination of WTGyrA+WTGyrB (A), GyrB-Asp464Asn (B), GyrB-Asn502Asp (C) and GyrB-Glu504Val (D). R and SC denote relaxed and supercoiled pBR322 DNA, respectively.

FQs-inhibited DNA supercoiling activities were assessed for the determination of IC_50_s. Figure 3 shows a representative result of the inhibitory effect of OFX and the results for the other FQs are presented in Figure S3. Results show the dose-dependent inhibition of five FQs against WT and mutant DNA gyrases, as summarized in Table 2. The five FQs inhibited the DNA supercoiling activities of WT DNA gyrase at low concentration (Table 2).

**Table 2:** IC_50_s and CC_25_s of FQs against WT and mutant DNA gyrases.

Drug	IC_50_ (µg/ml)				CC_25_ (µg/ml)			
	WT	Asp464Asn	Asn502Asp	Glu504Val	WT	Asp464Asn	Asn502Asp	Glu504Val
OFX	5.7±0.8	53.9±9.0	106.6±25.1	34.6±4.3	2.4±0.2	32.7±6.3	78.2±12.6	30.0±7.9
MXF	1.7±0.3	4.1±0.4	17.8±2.6	13.9±0.6	0.6±0.0	3.3±0.9	15.3±2.6	9.6±1.7
SIT	0.5±0.1	1.8±0.3	1.6±0.6	1.7±0.2	0.2±0.0	0.9±0.0	1.0±0.2	0.7±0.1
CIP	2.3±0.3	11.3±2.7	257.9±46.1	49.3±9.4	0.9±0.2	6.5±0.6	42.5±13.6	24.7±0.5
LVX	4.5±0.3	32.9±3.2	46.8±1.1	19.9±2.9	1.4±0.1	18.6±4.9	51.7±10.6	9.3±0.7

### CC_25_s of five FQs for WT and mutant DNA gyrases

**Figure 4:**
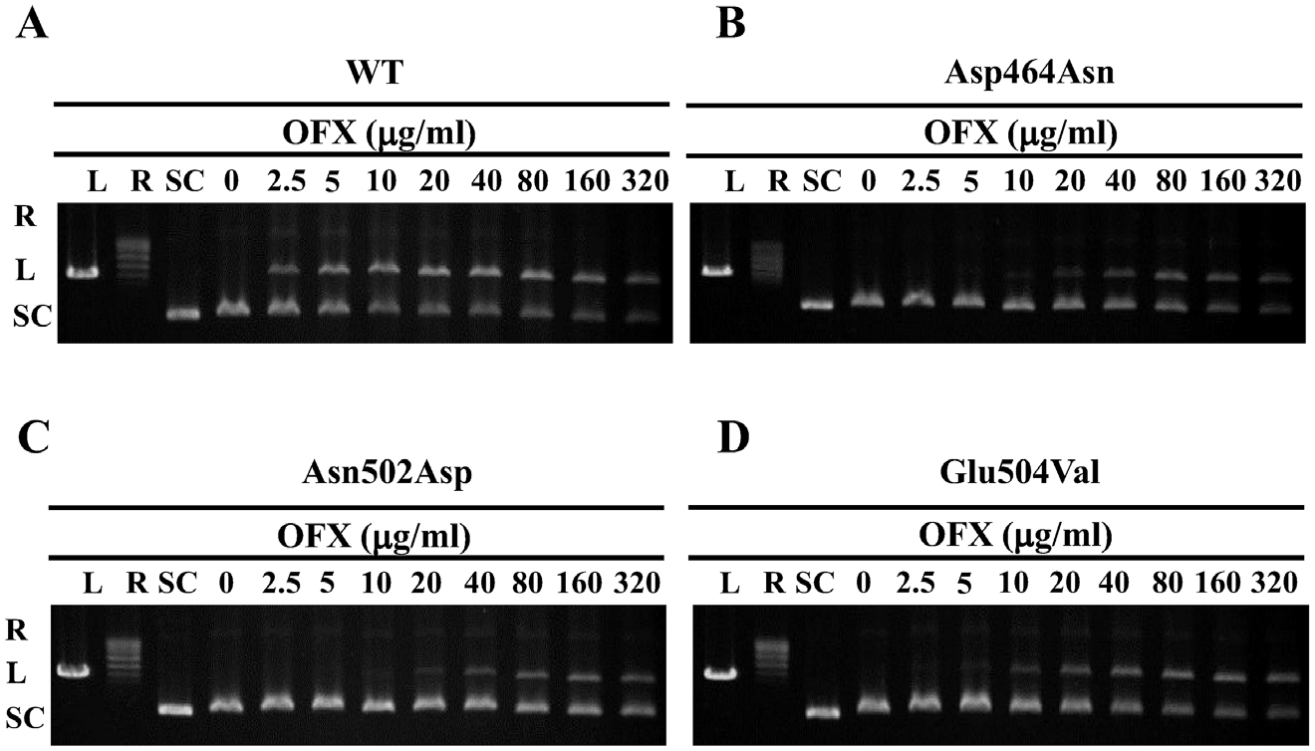
OFX-mediated DNA cleavage assay. Supercoiled pBR322 (0.3 mg) was incubated with GyrA (50 ng) and GyrB (50 ng) in the presence of the indicated concentration of OFX. DNA cleavage assay was performed in combination of WT GyrA+WT GyrB (A), GyrB-Asp464Asn (B), GyrB-Asn502Asp (C) and GyrB-Glu504Val (D). R, L and SC denote relaxed, linear and supercoiled pBR322 DNA, respectively.

DNA cleavage assay was performed in the presence of increasing concentrations of FQs to estimate CC_25_s. Figure 4 presents the results of a representative DNA cleavage assay using OFX, and Figure S4 shows those using other FQs. Table 2 shows the CC_25_s of FQs for WT and mutant DNA gyrases. Highest CC_25_s of FQs were observed for GyrB-Asn502Asp DNA gyrase.

## Discussion

**Figure 5:**
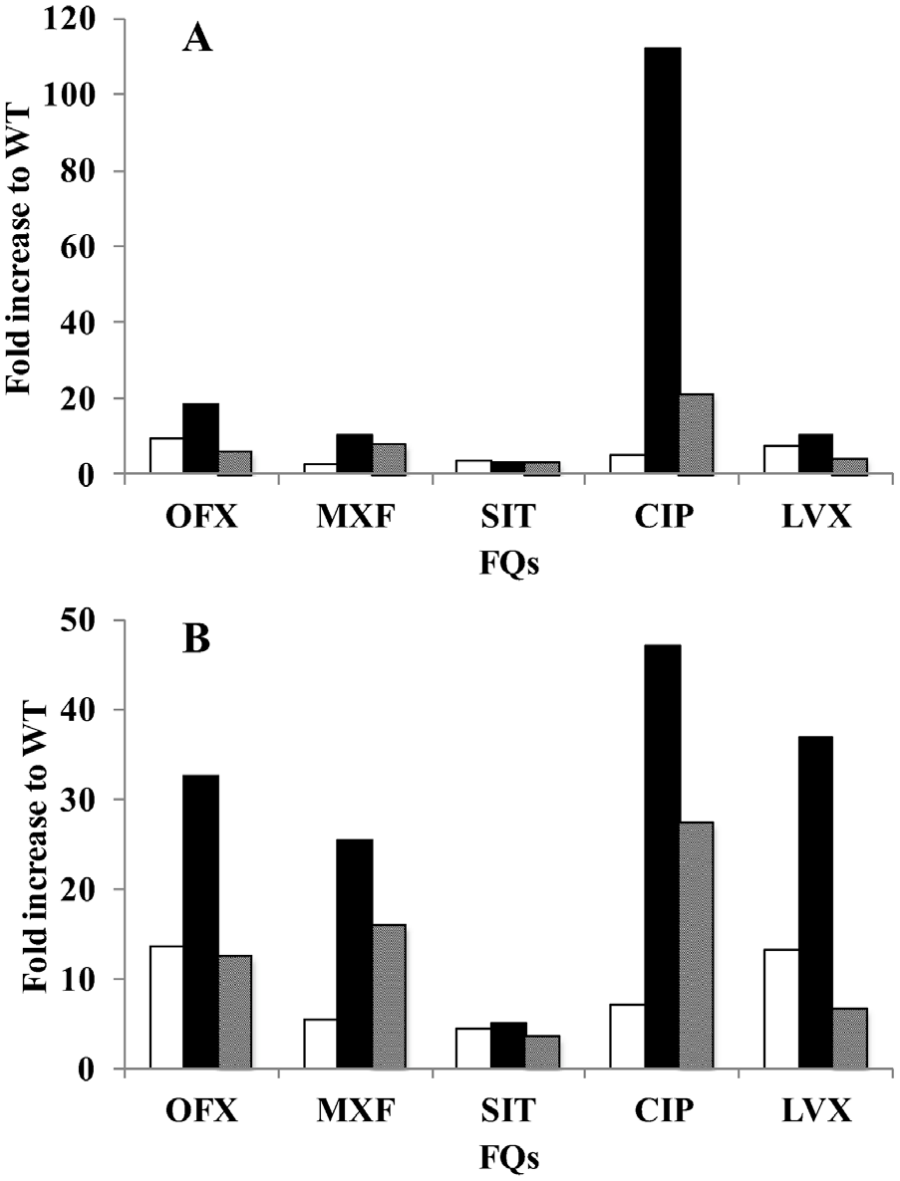
Increased IC_50_s and CC_25_s of FQs for mutant DNA gyrases. IC_50_s and CC_25_s were calculated by the quinolone-inhibited supercoiling assay and FQ-mediated cleavage assay, respectively. Fold increase of each FQ for mutant DNA gyrases was plotted. (A) IC_50_s, (B) CC_25_s. Open, closed and hatched bar denotes the value for GyrB- GyrB-Asp464Asn, GyrB-Asn502Asp and GyrB-Glu504Val DNA gyrase, respectively.

We focused on amino acid substitutions at position 464, 502 and 504 in GyrB in *M. leprae* equivalent to 461, 499 and 501, respectively, in *M. tuberculosis*, as amino acid substitutions at these positions in *M. tuberculosis* are known to contribute to FQ resistance [13]–[16], [20], [22], [23]. We carried out a FQ-mediated supercoiling activity inhibition assay and a DNA cleavage assay using recombinant WT and mutant DNA gyrases at 30°C, the optimal temperature of *M. leprae* growth [24], and calculated IC_50_s and CC_25_s of five FQs, including OFX, MXF, SIT, CIP and LVX. All FQs inhibited DNA supercoiling activities of WT DNA gyrase at low concentration (Table 2). In strong contrast, three mutant DNA gyrases showed reduced sensitivity to all five FQs. GyrB-Asn502Asp DNA gyrase exhibited the lowest FQ sensitivity among the three mutant DNA gyrases. IC_50_s of OFX, MXF, SIT, CIP and LVX for GyrB-Asp464Asn, Asn502Asp and Glu504Val DNA gyrases were 2.4- to 9.5-fold, 3.2- to 112.1-fold and 3.4- to 21.4-fold higher than those for WT DNA gyrase (Figure 3, 5, S3 and Table 2). A similar tendency was observed in the DNA cleavage assay. Namely, CC_25_s of OFX, MFX, SIT, CIP and LVX for GyrB-Asp464Asn, Asn502Asp and Glu504Val DNA gyrases were 4.5- to 13.6-fold, 5.0- to 47.2-fold and 3.5- to 27.4-fold higher than for WT DNA gyrase (Figure 4, 5, S4, Table 2). These results suggested the contribution of these amino acid substitutions in GyrB to reduced FQ sensitivity and the possible emergence of *M. leprae* with mutant GyrB, although previously identified Asp to Asn amino acid substitution in GyrB at position 205 [8] was revealed not to have an effect on FQ susceptibility [13]. It is noteworthy that mutant DNA gyrases exhibited a similar sensitivity pattern to those reported for mutant GyrB in *M. tuberculosis*. *M. leprae* GyrB-Asn502Asp DNA gyrase had lower FQ sensitivity than GyrB-Asp426Asn and GyrB-Glu504Val DNA gyrase, as has been shown in *M. tuberculosis* [15]–[18]. The high homology of the entire GyrB and full sequence match in QRDR between *M. leprae* and *M. tuberculosis* might lead to a similar tendency of FQ sensitivity. It is interesting that the Asp to Asn amino acid substitution in *E. coli* at position equivalent to 464 in *M. leprae* showed enhancing effect on CIP resistance [25] where Glu to Asp or Ala amino acid substitution in *Streptococcus pneumonia* at position equivalent to 504 in *M. leprae* showed little or reducing effect on CIP resistance, respectively [26]. Overall or QRDR structure of GyrB might affect the acquisition of FQ resistance.

IC_50_s of FQs were 8 to 40 times higher than the minimum inhibitory concentrations (MICs) in *M. tuberculosis* [17], [18], [22]. This non-proportionality presumably reflects basic differences in the cell-permeating properties and the accumulation of different FQs [22]. We investigated the inhibitory effects of OFX, GAT, MXF, LVX and SIT against WT and mutant DNA gyrases. IC_50_s of OFX for WT DNA gyrase was 5.7 µg/ml (Table 2) and it seemed reasonable that OFX has been used by a single application of 400 to 600 mg for leprosy patients with a single lesion and two or three doses of 400 to 600 mg in combination with first-line drugs, DDS and RIF [27] for the treatment of patients with MDR leprosy. On the contrary, IC_50_s of OFX for GyrB-Asp464Asn, Asn502Asp and Glu504Val showed 9.5, 18.7 and 6.1 fold higher concentration comparing to WT DNA gyrase, respectively, and OFX seems not to have the ability to inhibit *M. leprae* with DNA gyrase with these mutations. On the other hand, the order of inhibitory activity was SIT>MXF>CIP>LVX>OFX. Namely, SIT most effectively inhibited WT and mutant DNA gyrases among five FQs. IC_50_s of SIT for WT was 0.5 µg/ml and the increase was 3.6-, 3.2- and 3.4-fold for GyrB-Asp464Asn, GyrB-Asn502Asp and GyrB-Glu504Val DNA gyrases, respectively. In addition, the maximum serum concentration (*C*max) of OFX, SIT, CIP and LVX in 100 mg dosage was determined in clinical trials to be 0.95, 1.00, 1.33 and 1.22 µg/ml, respectively [28]–[31], and that of MFX in 400 mg dose to be 4.13 [32]. SIT might strongly inhibit *M. leprae* carrying GyrB-Asp464Asn, Asn502Asp and Glu504Val DNA gyrase as well as that carrying GyrA-Ala90Val, Asp95Gly, and Asp95Asn [14], [16], [20]. Thus, SIT is a promising candidate for the treatment of leprosy caused by OFX-resistant *M. leprae* with these problematic gyrases. Although SIT is now only approved in Japan and mild gastrointestinal disorders as adverse reactions have been reported, our data in this study might encourage the use of SIT for OFX-resistant leprosy.

In conclusion, we revealed the contribution of Asp464Asn, Asn502Asp and Glu504Val amino acid substitution to reduced sensitivity to FQ in *M. leprae* by an *in vitro* assay. This suggested the possible emergence of FQ-resistant *M. leprae* carrying GyrB with these amino acid substitutions in the future. Hence we would like to propose the analysis of these amino acid substitutions in GyrB to detect FQ-resistant leprosy. Additionally, effectiveness of sitafloxacin to the mutant DNA gyrases suggested the potential use of this FQ for the treatment of ofloxacin resistant cases.

## Supporting Information

Figure S1 **SDS-PAGE analysis of purified** ***M. leprae*** **DNA gyrases.** The His-tagged recombinant DNA gyrases were over expressed in *E. coli* and purified by Ni-NTA affinity resin chromatography. Lanes: M: Protein marker (NEB), 1: WTGyrA, 2: WTGyrB, 3: GyrB-Asp464Asn, 4: GyrB-Asn502Asp, 5: GyrB-Glu504Val. 300 ng of each protein was loaded on 5–20% gradient polyacrylamide gel.
(TIF)

Figure S2 **DNA supercoiling assay.** Supercoiling activities of WT DNA gyrase (A), DNA gyrases bearing GyrB-Asp464Asn (B), Asn502Asp (C) and Glu504Val (D) were analyzed. Relaxed pBR322 (0.3 mg) was incubated with GyrA (50 ng) or GyrB (50 ng) or both. Lanes: 1: relaxed pBR322 alone, 2: relaxed pBR322 and ATP, 3: relaxed pBR322, ATP, GyrA and GyrB, 4: relaxed pBR322, ATP and GyrA, 5: relaxed pBR322, ATP and GyrB, 6: relaxed pBR322, GyrA and GyrB.
(TIF)

Figure S3 **Inhibitory activities of (A) MXF, (B) SIT, (C) CIP and (D) LVX on supercoiling activities against** ***M. leprae*** **WT and mutant DNA gyrases.** Relaxed pBR322 DNA (0.3 mg) was incubated with 50 ng each of GyrA and GyrB in the absence or presence of the indicated concentration (in mg/ml) of three FQs. The reactions were stopped, and the DNA products were analyzed by electrophoresis on 1% agarose gel. R and SC denote relaxed and supercoiled pBR322 DNA, respectively.
(TIF)

Figure S4 **DNA cleavage activity of (A) MXF, (B) SIT, (C) CIP and (D) LVX against** ***M. leprae*** **WT and mutant DNA gyrases.** Supercoiled pBR322 DNA (0.3 mg) was incubated with 50 ng each of GyrA and GyrB in the absence or presence of the indicated concentration (in mg/ml) of three FQs. The reactions were stopped, and the processed DNA products were analyzed by electrophoresis on 1% agarose gel. R, L and SC denote relaxed, linear and supercoiled pBR322 DNA, respectively.
(TIF)
