# Supplementary figures and images for: Impact of Amino Acid Substitutions in B Subunit of DNA Gyrase in Mycobacterium leprae on Fluoroquinolone Resistance
Source: PLoS Negl Trop Dis. 2012 Oct 11;6(10):e1838. doi: 10.1371/journal.pntd.0001838 (PMC3469482; doi:10.1371/journal.pntd.0001838)

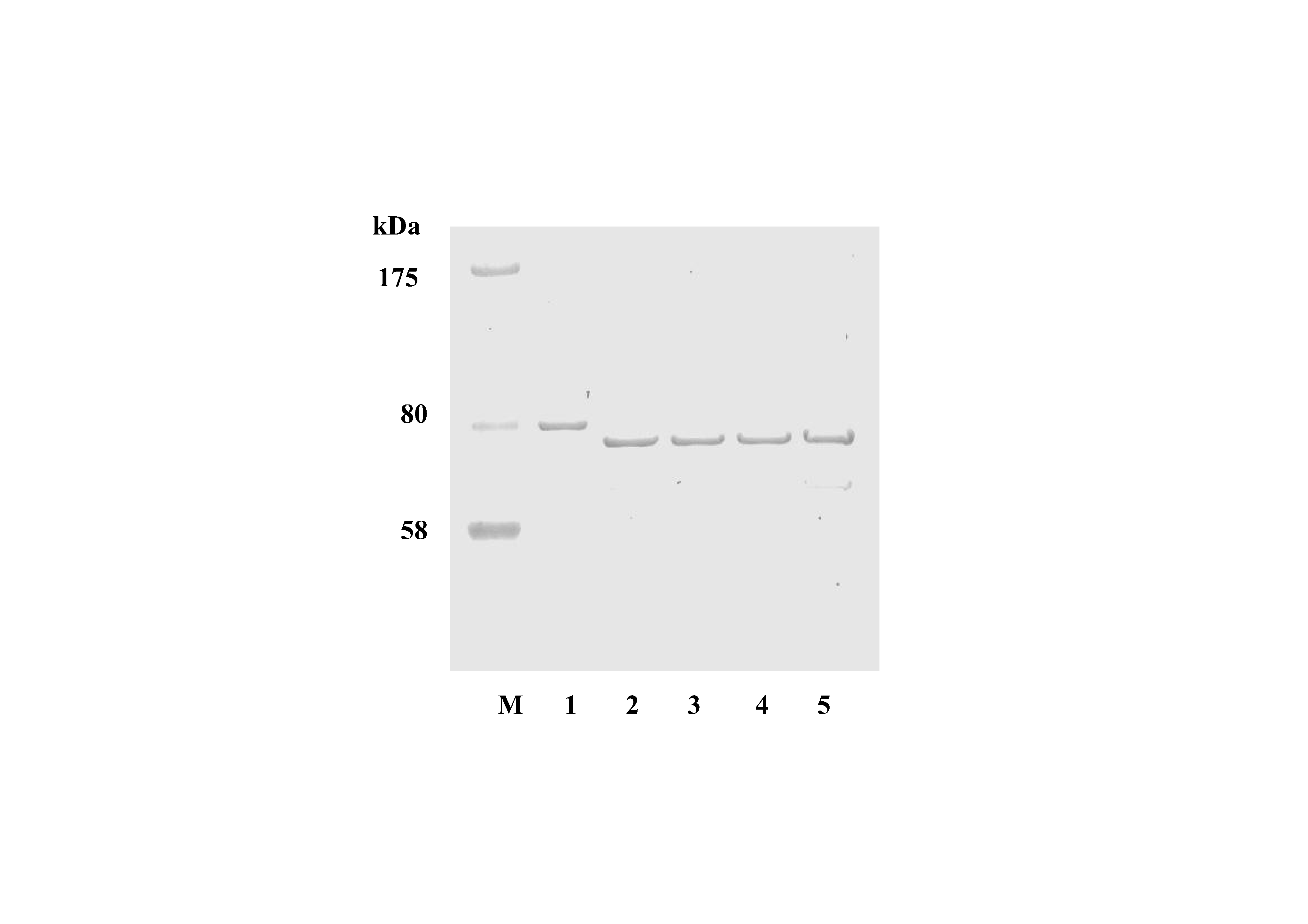

Supplement: Figure S1 — SDS-PAGE analysis of purified M. leprae DNA gyrases. The His-tagged recombinant DNA gyrases were over expressed in E. coli and purified by Ni-NTA affinity resin chromatography. Lanes: M: Protein marker (NEB), 1: WTGyrA, 2: WTGyrB, 3: GyrB-Asp464Asn, 4: GyrB-Asn502Asp, 5: GyrB-Glu504Val. 300 ng of each protein was loaded on 5–20% gradient polyacrylamide gel. (TIF) [file pntd.0001838.s001.tif]

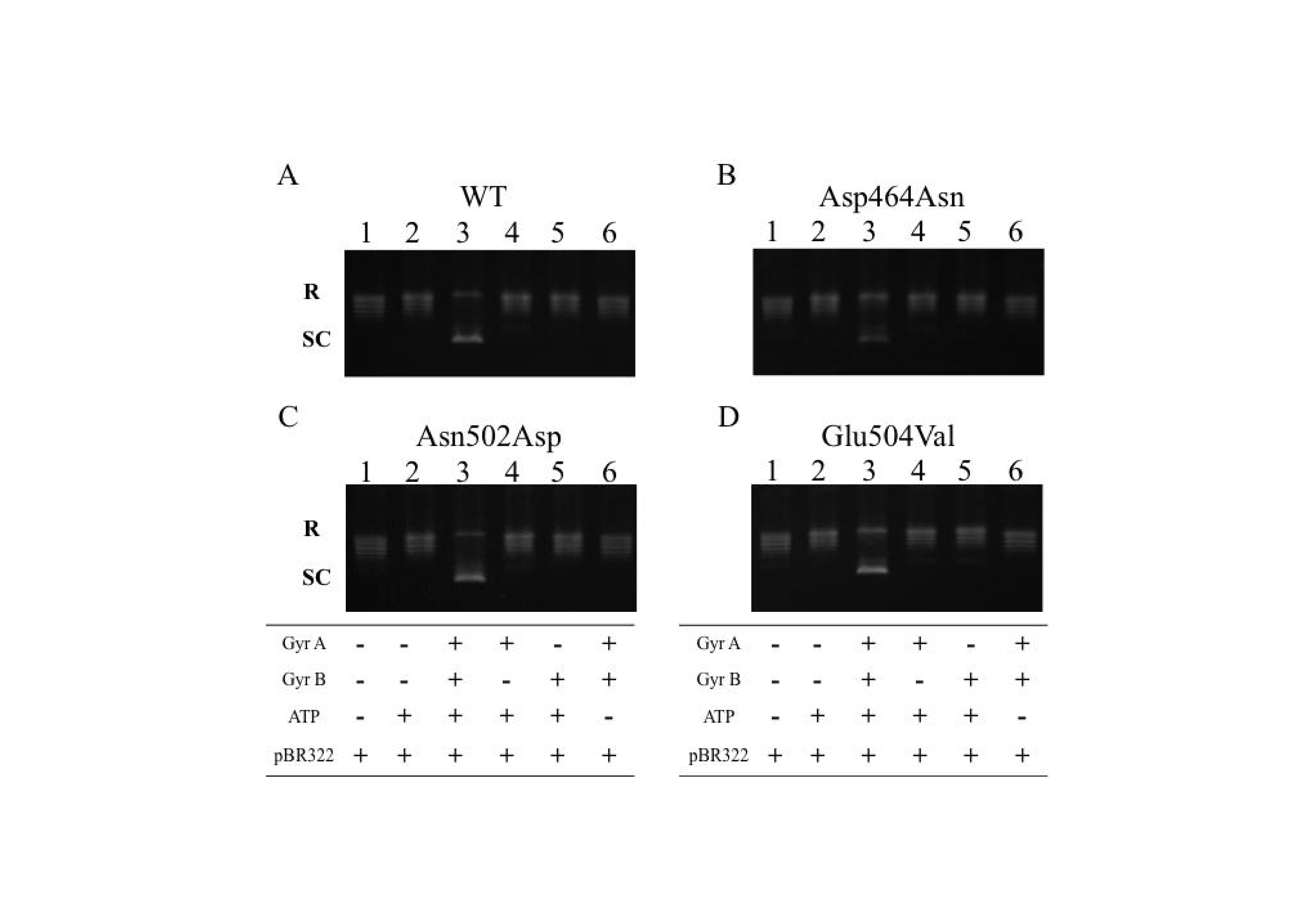

Supplement: Figure S2 — DNA supercoiling assay. Supercoiling activities of WT DNA gyrase (A), DNA gyrases bearing GyrB-Asp464Asn (B), Asn502Asp (C) and Glu504Val (D) were analyzed. Relaxed pBR322 (0.3 mg) was incubated with GyrA (50 ng) or GyrB (50 ng) or both. Lanes: 1: relaxed pBR322 alone, 2: relaxed pBR322 and ATP, 3: relaxed pBR322, ATP, GyrA and GyrB, 4: relaxed pBR322, ATP and GyrA, 5: relaxed pBR322, ATP and GyrB, 6: relaxed pBR322, GyrA and GyrB. (TIF) [file pntd.0001838.s002.tif]

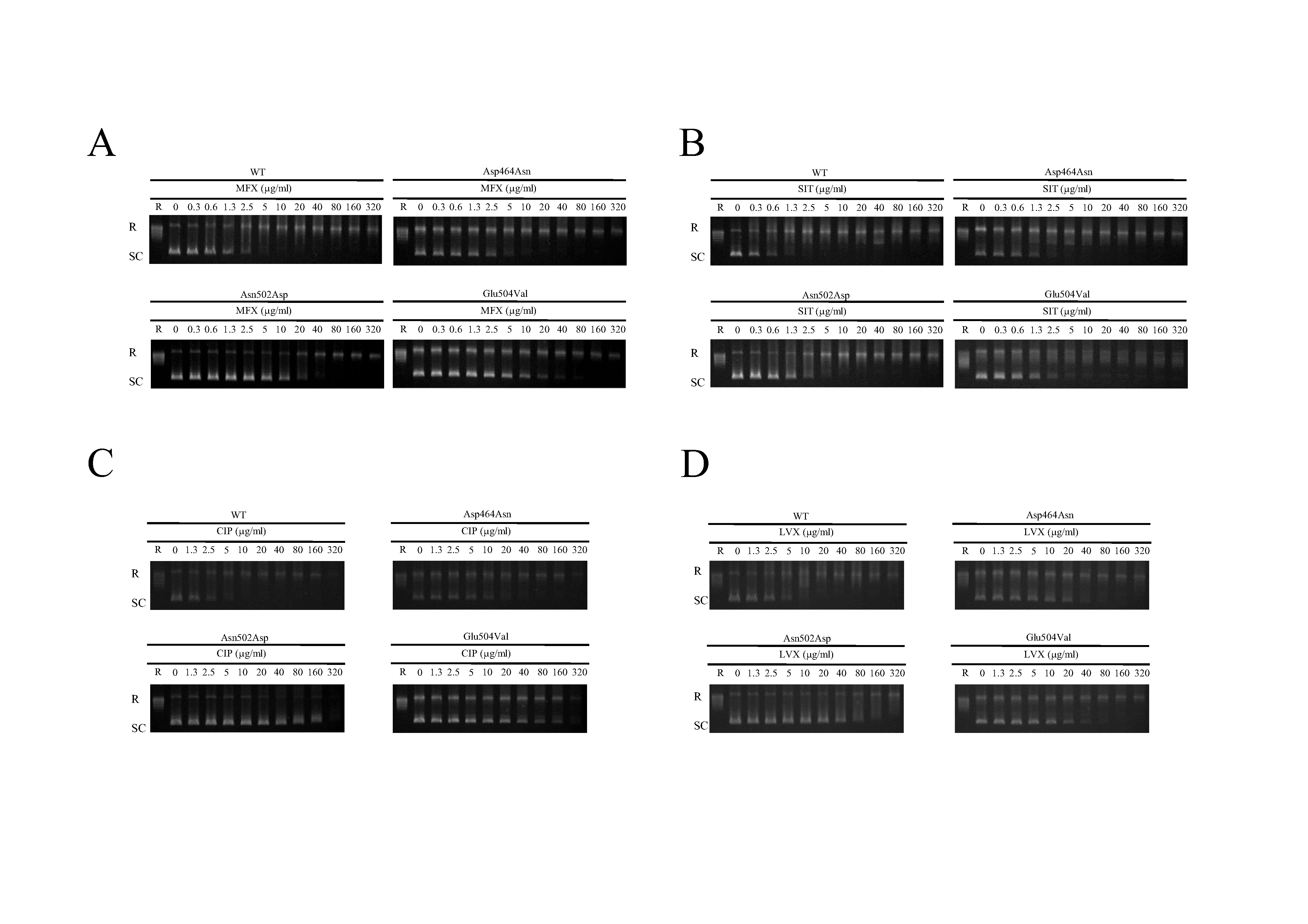

Supplement: Figure S3 — Inhibitory activities of (A) MXF, (B) SIT, (C) CIP and (D) LVX on supercoiling activities against M. leprae WT and mutant DNA gyrases. Relaxed pBR322 DNA (0.3 mg) was incubated with 50 ng each of GyrA and GyrB in the absence or presence of the indicated concentration (in mg/ml) of three FQs. The reactions were stopped, and the DNA products were analyzed by electrophoresis on 1% agarose gel. R and SC denote relaxed and supercoiled pBR322 DNA, respectively. (TIF) [file pntd.0001838.s003.tif]

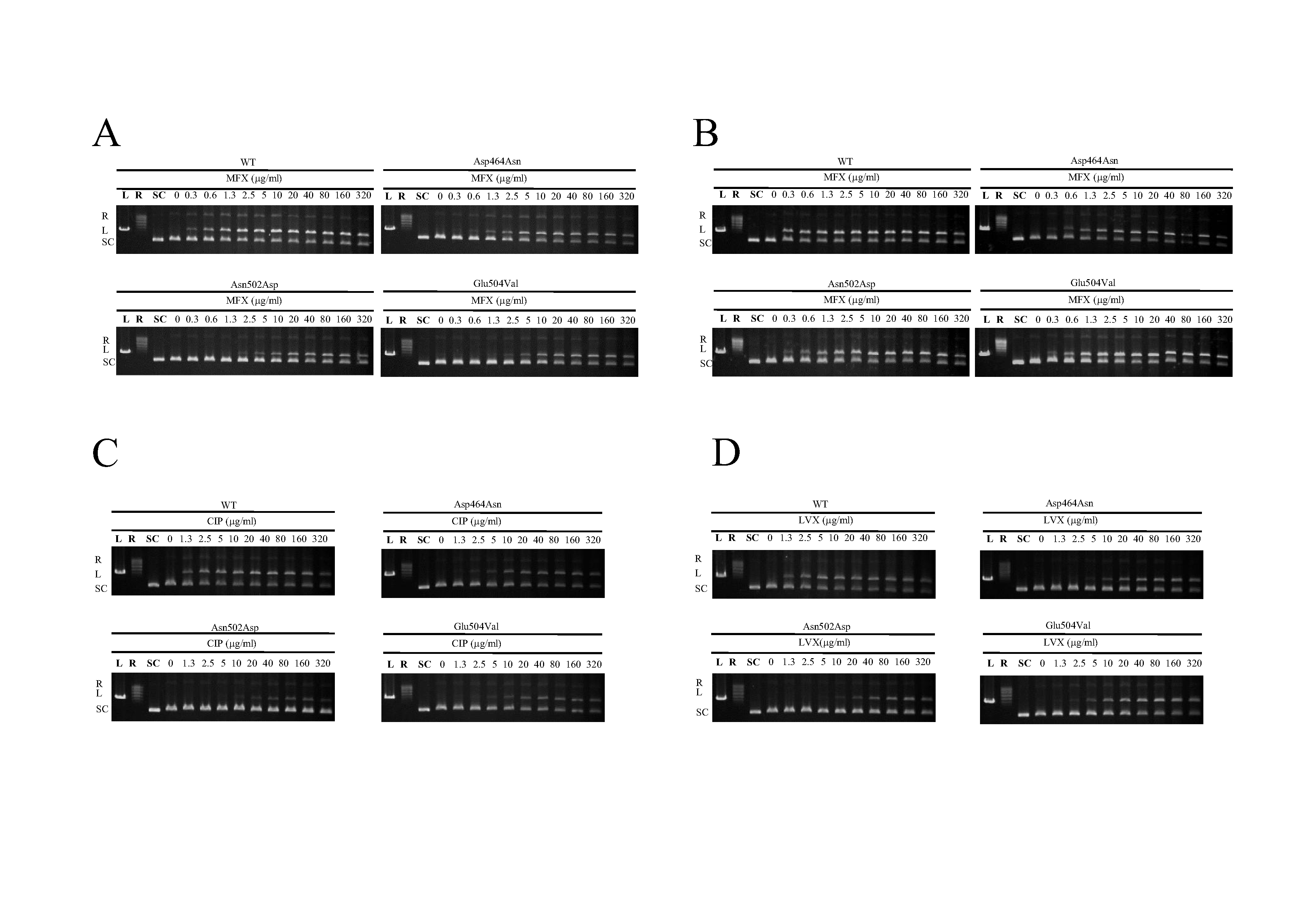

Supplement: Figure S4 — DNA cleavage activity of (A) MXF, (B) SIT, (C) CIP and (D) LVX against M. leprae WT and mutant DNA gyrases. Supercoiled pBR322 DNA (0.3 mg) was incubated with 50 ng each of GyrA and GyrB in the absence or presence of the indicated concentration (in mg/ml) of three FQs. The reactions were stopped, and the processed DNA products were analyzed by electrophoresis on 1% agarose gel. R, L and SC denote relaxed, linear and supercoiled pBR322 DNA, respectively. (TIF) [file pntd.0001838.s004.tif]
